# CircMGA Depresses Myoblast Proliferation and Promotes Myotube Formation through miR-144-5p/FAP Signal

**DOI:** 10.3390/ani12070873

**Published:** 2022-03-30

**Authors:** Zhijun Wang, Min Zhang, Kan Li, Yangfeng Chen, Danfeng Cai, Biao Chen, Qinghua Nie

**Affiliations:** 1 Department of Animal Genetics, Breeding and Reproduction, College of Animal Science, South China Agricultural University, Guangzhou 510642, China; zhijunwang@scau.edu.cn (Z.W.); zhangmin@hanbio.net (M.Z.); likan@stu.scau.edu.cn (K.L.); chenyangfeng@stu.scau.edu.cn (Y.C.); dfcai@stu.scau.edu.cn (D.C.); 2 National-Local Joint Engineering Research Center for Livestock Breeding, Guangdong Provincial Key Lab of Agro-Animal Genomics and Molecular Breeding and Key Laboratory of Chicken Genetics, Breeding and Reproduction, Ministry of Agriculture, Guangzhou 510642, China; 3 College of Animal Science and Technology, Jiangxi Agricultural University, Nanchang 330045, China

**Keywords:** myogenesis, chicken, circMGA, miR-144-5p, FAP, competing endogenous RNA

## Abstract

**Simple Summary:**

Circular RNAs (circRNAs) are a class of RNAs with a circular structure that can regulate genes by acting as microRNA sponges to neutralize microRNA and release mRNA. Poultry muscle growth and development include the increase of myoblast cell numbers before birth and hypertrophy of muscle fibers after birth. In this study, we used a series of molecular and cell biology methods to identify a novel circRNA, named circMGA, that could combine miR-144-5p competitively with FAP and inhibit myoblast proliferation and promote myotube formation. The results of this study have provided new evidence and extended the knowledge about non-coding RNAs in muscle development.

**Abstract:**

Circular RNAs are endogenous and abundant in skeletal muscle, and may not only be involved in regulating gene expression in a variety of ways, but also function as important regulators in poultry muscle development. Our previous research found that circMGA was differentially expressed during chicken muscle embryo development; however, as a novel circular RNA, the regulating mechanism of circMGA in myogenesis has never been studied before. In this study, we aimed to investigate the functional roles and related molecular mechanisms of circMGA in chicken primary myoblast cells. CircMGA originated from the exon 13–14 of MGA gene, was differentially expressed during embryo development and myogenesis differentiation, and could inhibit myoblast cell proliferation by repressing cell cycle related genes and promote myotube formation through MyoD and MyHC. Biotin-labeled miRNA pulldown assay and luciferase reporter assay result showed that miR-144-5p could directly target circMGA and FAP, indicating that there could be a competing endogenous RNA mechanism between circMGA and FAP. In function, miR-144-5p showed opposite regulation in myoblast cell with circMGA and FAP, just as expected. circMGA co-transfected with miR-144-5p or si-FAP could effectively eliminate the inhibition of miR-144-5p on myoblast proliferation and differentiation. In conclusion, we found a novel circRNA, named circMGA, which generated from the 13–14 exon of the MGA gene, and could inhibit myoblast proliferation and promote myotube formation by acting as the sponge of miR-144-5p and through miR-144-5p/FAP signal. Moreover, circMGA could effectively eliminate the inhibition of miR-144-5p on myoblast differentiation, thus releasing FAP and promoting myotube formation.

## 1. Introduction

Circular RNAs (circRNAs) are mainly formed from back splicing and have critical roles in gene regulation by acting as the sponge to neutralize microRNA (miRNA); they normally are considered as a new class of non-coding RNAs with no polyadenylated (polyA) tails [1,2,3]. Due to their circular structure, they are more stable than other RNAs, resistant to RNase R digest and other exonucleases, and have a longer half-life which could cause the accumulation of circRNAs [4,5,6]. Despite miRNA sponge, circRNA could also act as protein sponges [7,8] or encode proteins [9,10] or other unknown functions.

Myogenesis is a complex process involving multiple factors including mainly regulated genes and some non-coding RNAs [11,12,13]. Research has found that circRNAs were abundant in skeletal muscle development and the number of found circRNAs ranged from 1500 to 38,000 between species and most of their discovered mechanism in myogenesis were as miRNA sponge [14]. Since most studied circRNAs were from exon and shared most of the nucleotides with their parent genes except junction site, it is easy for them to possess abundant binding sites for miRNAs. The competing endogenous RNA (ceRNA) says that at the posttranscriptional level, mRNA, long non-coding RNAs (lncRNAs), and circRNA could regulate the target downstream genes through competing binding to the same miRNA [15,16,17,18]. miRNAs were known as mRNA inhibitors by targeting the untranslated region (UTR) of their mRNAs with argonaute protein [19,20]. It has been reported that some miRNA families like miR-1 and miR-133 family were myogenic specific and could regulate myogenesis [21,22]. miR-144-5p as a member of miR-144, could target RANKL and suppress cytokines expression [23] and involve in circ_0006282/miR-144-5p/YWHAB axis [24], but no research has reported its role on myogenesis.

Our previous study on chicken leg muscle at the age of 11 and 16 embryo day (E11, E16), and 1 day post hatch (P1) found more than 400 differentially expressed circRNAs, and circMGA is one of them [25]. The parent gene of circMGA is MGA, also named MAX (MYC associated factor X) dimerization protein (MGA), which consists of 26 exons. CircMGA is not only differentially expressed during embryo muscle growth, but also in different stages of myoblast differentiation. We used RNAhybrid to predict that circMGA and FAP contain the binding site for miR-144-5p. Fibroblast activation protein alpha (FAP) is a type II integral membrane protein belonging to the dipeptidyl peptidase 4 protein family and is famous for its high expression in tumor stroma [26]. Research has mainly focused on the dipeptidyl peptidase activity and fibrinolysis of FAP, but seldom in myogenesis. Therefore, in this study, we aimed to analyze the function of circMGA, miR-144-5p, and FAP in myogenesis and validated their ceRNA mechanism, which is circMGA/miR-144-5p/FAP signal.

## 2. Materials and Methods

### 2.1. Primers, Vector Construction and RNA Oligonucleotides

All primers used in this study were designed by Premier Primer 5.0 software, synthesized by Tsingke (Tsingke Biotech, Beijing, China), and listed in Table 1. The full length of circMGA and FAP were cloned into pCD25-ciR and pCD3.1 vector, respectively, for overexpression. The wide-type sequence of circMGA and FAP containing miR-144-5p binding sites were artificially synthesized by GeneCreat Biological Engineering (GeneCreat Biological Engineering, Wuhan, China) and cloned into the pmirGLO vector along with the mutation sequence respectively. The RNA oligonucleotide sequences and small interference RNA (siRNA) sequences used in this study were listed in Table 2.

### 2.2. Cell Culture and Transfection

Chicken primary myoblast cell isolation: leg muscle tissues were harvested from 11-day-old chicken embryos (Zhuhai Yuhe Company, Guangdong, China); skin and bones were removed and digested in 0.25% trypsin for 10 min. Complete growth medium (RPMI-1640 medium with 20% fetal bovine serum and 0.5% penicillin/streptomycin (Gibco, Carlsbad, CA, USA)) was used to neutralize the slurry, and the slurry was then passed twice through 70- and 40-μm filters, and centrifuged for 5 min at 500× *g*. Differential velocity adherent method (40 min, twice) was used here to remove fibroblasts, and then keep the supernatant and seed in 100 mm dishes for chicken primary myoblast cell culture.

Chicken primary myoblast cells were seeded at a density of 2 × 10^4^ cells/cm^2^ on 12 well culture plates for 12 h, and the plasmid (1 μg) or siRNA (100 nM) transfection was performed with Lipofectamine 3000 reagent (Invitrogen, Carlsbad, CA, USA) following the manufacturer’s protocol with at least three replications.

### 2.3. RNA Exaction, cDNA Synthesis and Quantitative Real-Time PCR (qRT-PCR)

Total RNA was extracted using the chloroform isopropanol method with RNAiso Plus reagent (Takara, Otsu, Japan). As for RNase R treatment, 10U RNase R was used to digest 2.5 μg total RNA at 37 °C for 30 min and employed to synthesize cDNA for qRT-PCR. cDNA synthesis for miRNA was performed with ReverTra Ace qPCR RT Kit (Toyobo, Osaka, Japan) using specific Bulge-loop miRNA qRT-PCR for miR-144-5p and U6 designed by Ribobio (Ribobio, Guangzhou, China). The reverse transcription reaction for mRNA was performed with a HiScript II Q RT SuperMix for qPCR (+gDNA wiper) (Vazyme, Nanjing, China). qRT-PCR reactions were carried out in a QuantStudio 5 Real-Time PCR Systems (Thermo Fisher, Waltham, MA, USA) with iTaq^TM^ Universal SYBR Green Supermix (Bio-Rad, Hercules, CA, USA). The mRNA relative expression was calculated by using the 2(-Delta Delta C(T)) method [27].

### 2.4. Flow Cytometry Analysis

After 48 h transfection, the primary myoblast cell was collected and fixed in 70% ethanol and kept overnight at −20 °C. The cells were incubated with 50 μg/mL propidium iodide (Sigma, Louis, MO, USA), 10 μg/mL RNase A (Takara, Otsu, Japan), and 0.2% Triton X-100 (Sigma, Louis, MO, USA) for 30 min at 4 °C. The flow cytometry analysis was performed with a BD AccuriC6 flow cytometer (BD Biosciences, San Jose, CA, USA) and FlowJo (v7.6) software (Treestar Incorporated, Ashland, OR, USA).

### 2.5. EdU (5-Ethynyl-2′-Deoxyuridine) Assay

The myoblasts were incubated in 50 μM 5-ethynyl-2′-deoxyuridine for 2 h after 46 h transfection and then followed with a C10310 EdU Apollo In Vitro Imaging Kit (Ribobio, Guangzhou, China). All images were captured with a Leica DMi8 fluorescent microscope (Leica, Wetzlar, Germany) in 200X with 6 random fields in 3 wells per group, the proliferation rate = $\frac{EdU\;positive\;cells}{total\;Hoechst\;33,342\;stained\;cells}$.

### 2.6. Immunofluorescence

When the myoblast cells reached 90% influence, the medium was replaced with a differentiation medium (RPMI-1640 medium with 5% horse serum and 0.5% penicillin/streptomycin) and changed every day for 3 days. The myoblasts were fixed with 4% formaldehyde and blocked in 0.2% Triton-X 100 and 5% horse serum with PBS for 30 min, and incubated overnight with MyHC (1:50, Developmental Studies Hybridoma Bank) primary antibody, followed with goat anti-mouse IgG (H + L)-Dylight 594 (1:200, BS10027; Bioworld, Minneapolis, MN, USA) and Hoechst 33342 (1 mg/mL, H1399, Invitrogen, Carlsbad, CA, USA) for 1 h. All images were captured with a Leica DMi8 fluorescent microscope (Leica, Wetzlar, Germany) in 200X with 6 random fields in 3 wells per group, and the percentage of myotube area were calculated by ImageJ software (National Institutes of Health, Bethesda, MD, USA).

### 2.7. Dual-Luciferase Reporter Assay

Four co-transfection experiment groups were designed to test the binding relationship between circMGA/FAP with miR-144-5p, which were pmirGLO-circMGA/FAP-WT + miR-144-5p mimic, pmirGLO-circMGA/FAP-WT + mimic NC, pmirGLO-circMGA/FAP-MT + miR-144-5p mimic, and pmirGLO-circMGA/FAP-MT + mimic NC. Firefly and Renilla luciferase activities were measured with a Dual-Glo Luciferase Assay System Kit (Promega, Madison, WI, USA) in a Fluorescence/Multi-Detection Microplate Reader (BioTek, Winooski, VT, USA).

### 2.8. Biotin-Labeled miRNA Pulldown Assay

Transfection of 3′ end biotin-labeled miR-144-5p or mimic NC (100 nM) into myoblast cells in two 100 mm dishes was conducted, and the cell was harvested after 36 h transfection and lysed in lysis buffer. The cell lysates were incubated with streptavidin magnetic beads for 4 h at 4 °C and washed with lysis buffer five times before RNA extraction.

### 2.9. Statistical Analysis

Results were showed as mean ± S.E.M with 4–6 independent replications. An independent sample *t*-test was used to test the statistically significant difference between groups. We considered *p* < 0.05 to be statistically significant. * *p* < 0.05; ** *p* < 0.01; *** *p* < 0.001.

## 3. Results

### 3.1. CircMGA Differentially Expressed during Embryonic Development

**Figure 1:**
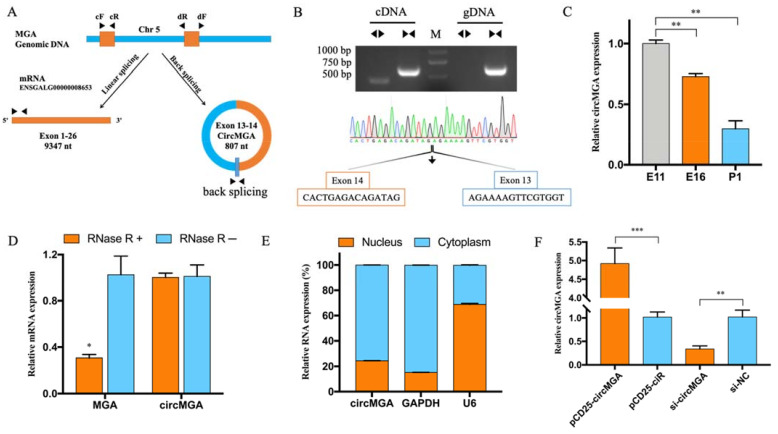
The characteristic of circMGA. (**A**) The schema of circMGA is derived from the exon 13-14 of the MGA gene. (**B**) Divergent primer amplified circMGA in cDNA but not gDNA, Sanger sequencing confirmed the junction sequence of circMGA. (**C**) The expression pattern of circMGA in E11, E16, and P1. (**D**) qRT-PCR results of MGA mRNA and circMGA after RNase R digestion. (**E**) CircMGA is localized mainly in the cytoplasm of myoblast cell. GAPDH and U6 serve as cytoplasmic and nuclear localization controls respectively. (**F**) CircMGA successfully overexpressed and inhibited in myoblast after transfected with pCD25-circMGA and si-circMGA. (* *p* < 0.05; ** *p* < 0.01, *** *p* < 0.001).

Previous circRNA sequencing (accession number GSE89355) on leg muscle at the age of 11 and 16 embryo day (E11, E16), and 1 day post hatch (P1) showed that circMGA was differentially expressed during chicken embryonic leg muscle development and the relative expression level was gradually decreased in these three periods (Figure 1C), as detected by qRT-PCR. CircMGA is formed by the cyclization of exon 13–14 of MGA, with a total length of 807 nt (Figure 1A). The PCR result amplified by convergent primer (cF/R) and divergent primer (dF/R) shows that divergent primer gets a single distinct band only in the cDNA sample along with the sanger sequencing matched to the back spliced sequence indicating the real existence of circMGA (Figure 1B, see supplementary materials File S1 for all complete agarose gel electrophoresis image). We also employed RNase R treatment to detect the resistance of circMGA and the result showed much more stability and resistance than the linear MGA mRNA (Figure 1D). The cellular localization of circMGA had also been studied and result showed mainly in the cytoplasm (Figure 1E). The efficiency of the overexpression vector of circMGA and siRNA was detected in myoblast cells for further study (Figure 1F).

### 3.2. CircMGA Depressed Myoblast Cell Cycle Progress, Promoted Myotube Formation, and Sponged miR-144-5p

**Figure 2:**
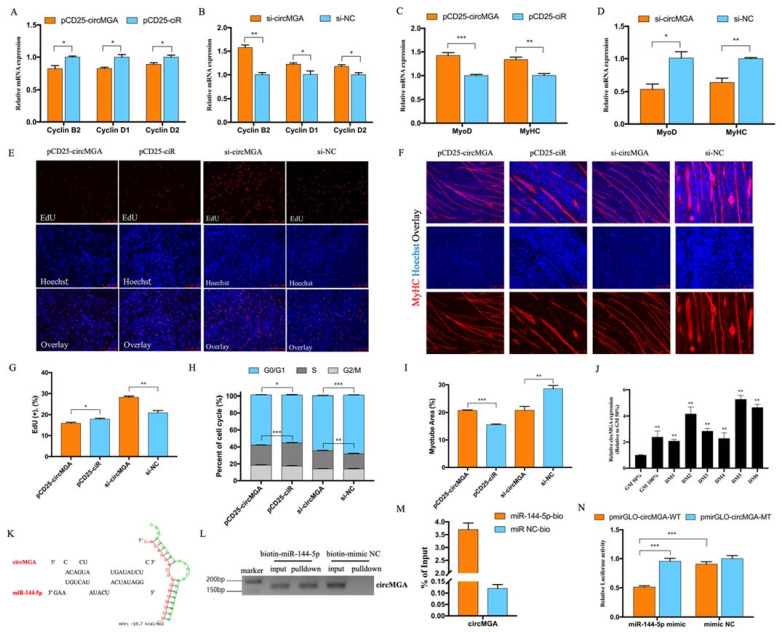
CircMGA depressed myoblast proliferation promoted myotube formation and sponged miR-144-5p. (**A**,**B**) qRT-PCR results of Cyclin B2, Cyclin D1, and Cyclin D2 after circMGA overexpression and knockdown. (**C**,**D**) qRT-PCR results of MyoD and MyHC after circMGA overexpression and knockdown. (**E**) EdU staining and the positive EdU cell rate (**G**) for myoblast cell after circMGA overexpression and knockdown. (**F**) MyHC immunofluorescence staining of myotube and myotube area percentage (**I**) after transfection. (**H**) Cell cycle analysis after circMGA overexpression and knockdown. (**J**) The expression pattern of circMGA in different stages of myoblast differentiation. GM (growth media) stands for myoblasts in the proliferative phase. DM (differentiation media), DM1-DM6 means differentiation from day 1 to day 6. (**K**) RNAhybrid software analysis the potential binding site between circMGA and miR-144-5p. (**L**,**M**) Biotin-labeled miRNA pulldown revealed the interaction between circMGA and miR-144-5p. (**N**) Relative luciferase activity of pmirGLO-circMGA-WT/MT plasmid with miR-144-5p mimic or mimic NC. (* *p* < 0.05; ** *p* < 0.01, *** *p* < 0.001).

To find out the role of circMGA in myoblast proliferation, we checked the expression of Cyclin B2, Cyclin D1, and Cyclin D2, and found out the overexpression of circMGA could inhibit cell cycling related genes (Figure 2A) while knockdown accelerates their expression (Figure 2B). Thus, we used flow cytometry to analyze the change of cell cycle after circMGA overexpression or inhibition; the number of cells that remained in the S phase significantly decreased after circMGA overexpression, and inhibition of circMGA showed the opposite trend (Figure 2H). Moreover, the EdU positive cells were less than the control group after circMGA transfection or significantly increased after circMGA knockdown (Figure 2E,G). All of those above results suggest that circMGA could function as a restrainer of myoblast. The qRT-PCR result of MyoD and MyHC expression (Figure 2C,D), as well as the expression pattern of circMGA during myoblast differentiation (Figure 2J), showed that circMGA may promote myoblast differentiation. The immunofluorescence of MyHC showed that the myotube area was largely increased after circMGA overexpression and significantly decreased after knockdown (Figure 2F,I). Numerous studies have reported that circular RNA achieved their function by acting as miRNA sponges, here we used RNAhybrid to predict the potential target miRNAs of circMGA and chose miR-144-5p as our candidate miRNA (Figure 2K). A biotin-labeled miRNA pulldown assay was performed here to confirm the interaction between circMGA and miR-144-5p; PCR results showed that compared with the biotin-mimic NC group, biotin-miR-144-5p could successfully pulldown circMGA (Figure 2L); and qRT-PCR results detected that there was about 3.5-fold enrichment of circMGA in the miR-144-5p labeled fragment (Figure 2M). We also constructed the recombinant pmirGLO vectors containing miR-144-5p binding site (pmirGLO-circMGA-WT) or binding site mutant (pmirGLO-circMGA-MT), and co-transfected with miR-144-5p mimic or mimic negative control (NC). The dual-luciferase reporter assay result showed that miR-144-5p significantly reduced the luciferase activity compared with the mutant group or mimic NC (Figure 2N), which proved the binding relationship of miR-144-5p and circMGA.

### 3.3. miR-144-5p Accelerated Myoblast Cell Cycle and Inhibited Myotube Formation by Targeting FAP

**Figure 3:**
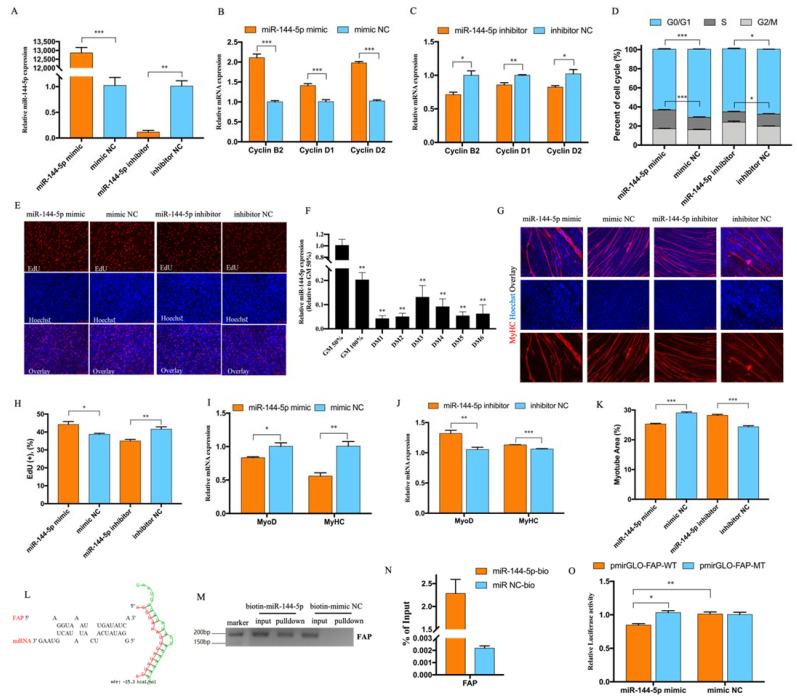
miR-144-5p accelerated the myoblast cell cycle and inhibited myotube formation by targeting FAP. (**A**) miR-144-5p successfully overexpressed and inhibited in myoblast after transfected with miR-144-5p mimic and inhibitor. (**B**,**C**) qRT-PCR results of Cyclin B2, Cyclin D1, and Cyclin D2 after miR-144-5p overexpression and knockdown. (**D**) Cell cycle analysis after miR-144-5p overexpression and knockdown. (**E**) EdU staining and positive EdU cell rate (**H**) for myoblast cell after miR-144-5p transfection. (**F**) The relative expression of miR-144-5p during myoblast differentiation. (**G**) MyHC immunofluorescence staining of myotube and myotube area percentage (**K**) after miR-144-5p transfection. (**I**,**J**) qRT-PCR results of MyoD and MyHC after miR-144-5p transfection. (**L**) RNAhybrid software analysis the potential binding site between miR-144-5p and FAP. (**M**,**N**) Biotin-labeled miRNA pulldown revealed the interaction between miR-144-5p and FAP. (**O**) The dual- luciferase reporter assay was performed by co-transfecting pmirGLO-FAP-WT/MT plasmid with miR-144-5p mimic or mimic NC. (* *p* < 0.05; ** *p* < 0.01, *** *p* < 0.001).

Considering circMGA could act as a miR-144-5p sponge, we also confirmed the function of miR-144-5p in myoblast cell after miR-144-5p mimic or inhibitor could successfully overexpress or interfered with the expression of miR-144-5p (Figure 3A). The expression of Cyclin B2, Cyclin D1, and Cyclin D2 was largely increased after miR-144-5p overexpression and decreased after inhibition (Figure 3B,C). Cell cycle analysis showed that miR-144-5p could promote cell cycle progress and increase the proportion of cells in the S phase (Figure 3D). EdU assay also proved that miR-144-5p could accelerate myoblast cell proliferation (Figure 3E,H). Interestingly, the expression of miR-144-5p in the myoblast differentiation stage was lower than the proliferation stage (Figure 3F), which was just the opposite of circMGA. The expression of MyoD and MyHC were inhibited (Figure 3I,J) as well as the myotube area after miR-144-5p overexpression and promoted after miR-144-5p knockdown (Figure 3G,K). To find the mechanism of miR-144-5p regulating myoblast proliferation and differentiation, we used RNAhybrid to predict the potential target of miR-144-5p and chose FAP as our target gene (Figure 3L). The biotin-labeled miRNA pulldown assay (Figure 3M,N) and the decreased luciferase activity of FAP (Figure 3O) showed that miR-144-5p could bind to FAP.

### 3.4. FAP Inhibited Myoblast Proliferation and Promote Myoblast Differentiation

**Figure 4:**
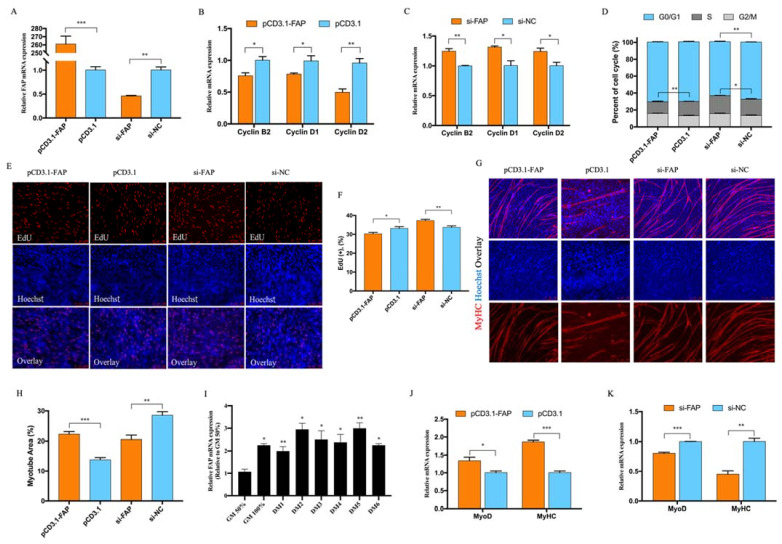
FAP inhibited myoblast proliferation and promote myoblast differentiation. (**A**) FAP successfully overexpressed and inhibited in myoblast after transfected with pCD3.1-FAP and si-FAP. (**B**,**C**) qRT-PCR results of Cyclin B2, Cyclin D1, and Cyclin D2 after FAP overexpression and knockdown. (**D**) Cell cycle analysis after FAP overexpression and knockdown. (**E**,**F**) EdU staining and positive EdU cell rate for myoblast cell after FAP transfection. (**G**,**H**) MyHC immunofluorescence staining of myotube and myotube area percentage after miR-144-5p transfection. (**I**) The relative expression of FAP during myoblast differentiation. (**J**,**K**) qRT-PCR results of MyoD and MyHC after FAP transfection. (* *p* < 0.05; ** *p* < 0.01, *** *p* < 0.001).

Our overexpression vector and siRNA of FAP were successfully caused over 200-fold overexpression and 50% knockdown (Figure 4A). FAP turns out to be more abundant during myoblast differentiation according to our qRT-PCR result (Figure 4I). The cell cycle progress and cell cycle related genes were significantly depressed with the existence of FAP and recovered after FAP knockdown as well as EdU positive cells (Figure 4B–F). The RNA expression of MyoD and MyHC (Figure 4J,K) were promoted after FAP overexpression as well as the myotube total area (Figure 4G,H). All of our results suggest FAP could inhibit myoblast proliferation and promote myotube formation.

### 3.5. CircMGA Promote Myotube Formation through miR-144-5p/FAP Signal

**Figure 5:**
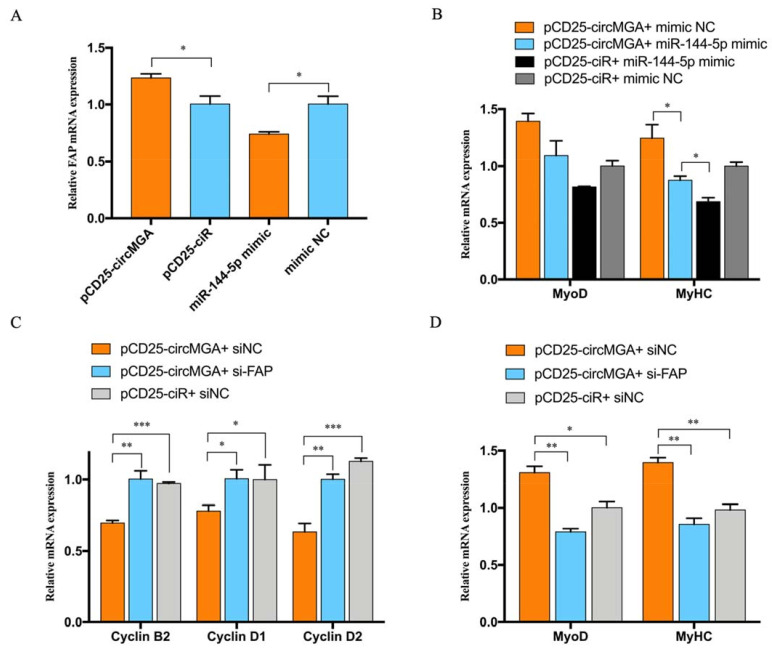
CircMGA promotes myotube formation through miR-144-5p/FAP signal. (**A**) The RNA expression of FAP after circMGA and miR-144-5p overexpression. (**B**) The mRNA expression of MyoD and MyHC after pCD25-circMGA and miR-144-5p co-transfection. (**C**) qRT-PCR results of Cyclin B2, Cyclin D1, and Cyclin D2 after pCD25-circMGA and si-FAP co-transfection. (**D**) Changes of MyoD and MyHC mRNA level after transfected with pCD25-circMGA and si-FAP/NC. (* *p* < 0.05; ** *p* < 0.01, *** *p* < 0.001).

To validate the competitive relationship between circMGA and FAP for miR-144-5p, we did a qRT-PCR to test the mRNA level of FAP after circMGA or miR-144-5p overexpression, and the result was the same, with the expectation that miR-144-5p could inhibit the expression of FAP, while the existence of circMGA could recover the inhibition of miR-144-5p to FAP and promote the expression of FAP (Figure 5A). Thus, we designed a co-transfection experiment to see whether miR-144-5p could stop the promoting effect of circMGA on the myoblast differentiation process. The result showed that the expression of MyoD and MyHC was neutralized and basically unchanged when co-transfected with circMGA and miR-144-5p compared to their single overexpression (Figure 5B). We also transfected circMGA with si-FAP, and the results showed that when FAP got knockdown, the extra miR-144-5p would bind to circMGA and offset the represses of circMGA on cell cycle related genes and promote the expression of MyoD and MyHC (Figure 5C,D). All of our results showed that circMGA could eliminate the inhibition effect of miR-144-5p on myotube formation, thus releasing FAP and promoting myoblast differentiation.

## 4. Discussion

The abundance of circRNAs and their stable structure determines their irreplaceable functional characteristics. CircRNAs have been found to be involved in many biological processes among species due to the development of high-throughput sequencing and have led to the discovery of a large number of circRNAs [28,29,30]. Here, we deeply analyzed our previous circRNA sequencing data and discovered a circRNA, named circMGA, that was differentially expressed during chicken muscle embryo development, and appears high expression level at DM2, DM5, and DM6 during myoblast differentiation (Figure 2J); this special expression pattern suggested that circMGA could have potential regulatory roles in myogenesis. As the parent gene of circMGA, MGA could negatively regulate cancer cell proliferation and act as an inhibitor for MYC target genes [31]; here in myoblast cells, circMGA shared the same repression on proliferation.

Myogenesis is a complex process, and myoblast proliferation and differentiation were two important parts of it. There are numerous genes, mainly myogenic regulatory factors (MRFs), that could regulate skeletal muscle differentiation, especially the MyoD family members (MyoD, Myf5, MyoG, and MRF4), and commonly, MyoD participated in myoblast determination and could regulate many myogenesis-related genes, thus promoting the formation of myotube [32,33,34]. MyHC is a myoblast differentiation marker gene, that acts as the muscle engine and the backbone of the sarcomere thick filaments [35]. Here we used qRT-PCR to monitor the effect of circMGA, miR-144-5p, and FAP on the expression of MyoD and MyHC, and results showed that circMGA and FAP could promote the expression of MyoD and MyHC. Thus, we concluded that the regulation of circMGA/miR-144-5p/FAP signal on the myogenic process is mainly through MyoD and MyHC.

The myoblast proliferation was promoted after miR-144-5p treatment, which was the opposite of what was found in gastric cancer cells, that miR-144-5p could inhibit the cell proliferation [24]; therefore, we believed that miR-144-5p promoted growth and displayed anti-cancer activities. As a member of the dipeptidyl peptidase 4 protein family, FAP has both dipeptidyl peptidase and endopeptidase activities, could modulate fibrinolysis, seems to mostly appear in many kinds of fibroblasts, and is essential for cell survival [26]. While in our study, the existence of FAP caused the inhibition of myoblast cell proliferation but promoted differentiation, the consistently high expression of FAP during myoblast differentiation also suggests the importance of FAP in myogenesis. However, besides MyoD and MyHC, whether FAP could have its enzyme activity or another regulator mechanism in myoblast cell still requires further investigation.

## 5. Conclusions

In conclusion, we found a novel circRNA, named circMGA, which generated from the 13-14 exon of the MGA gene, and could inhibit myoblast proliferation and promote myotube formation by acting as the sponge of miR-144-5p and through miR-144-5p/FAP signal. Moreover, circMGA co-transfected with miR-144-5p or si-FAP could effectively eliminate the inhibition of miR-144-5p on myoblast differentiation, thus releasing FAP and promoting myotube formation.

## Figures and Tables

**Table 1:** Primers used in this study.

Name	Nucleotide Sequences (5′-3′)	Application
d-circMGA	F: TCAGTACTGTGATCTCCAAGG	Divergent primer for circMGA
	R: ATGGCACTGAAATTTTCACTC	
c-MGA	F: CCTCATTATTGAATTGGCTTC	Convergent primer for MGA
	R: GTCTCAGTGGTGGGATAAGAG	
q-circMGA	F: TCCAAGGTAGCATCCAGTGC	qRT-PCR for circMGA
	R: TTCATTCCAGCTGCAGTCAGAG	
c-circMGA	F1: AGAAAAGTTCGTGGTCTTCCC	cloning full length primer of circMGA
	R1: CTATCTGTCTCAGTGGTGGGAT	
c-circMGA	F2: GGAATTCTAATACTTTCAGAGAAAAGTTCGTGGTCTTCCC	cloning full length primer of circMGA
	R2: CGGGATCCAGTTGTTCTTACCTATCTGTCTCAGTGGTGGGAT	
c-FAP	F: CGGGATCCATGAAGACCCGGCTAAAAGTA	cloning full CDS length primer of FAP
	R: GCTCTAGACTATTCTGACAAAGAAAAACATT	
q-FAP	F: GCTTCCGTCCAAAGTTGTCA	qRT-PCR for FAP
	R: TGTGCTGTTGGTCATGATGGTA	
Cyclin B2	F: CAGTAAAGGCTACGAAAG	qRT-PCR for Cyclin B2
	R: ACATCCATAGGGACAGG	
Cyclin D1	F: CAGAAGTGCGAAGAGGAAGT	qRT-PCR for Cyclin D1
	R: CTGATGGAGTTGTCGGTGTA	
Cyclin D2	F: AACTTGCTCTACGACGACC	qRT-PCR for Cyclin D2
	R: TTCACAGACCTCCAACATC	
MyoD	F: GCTACTACACGGAATCACCAAAT	qRT-PCR for MyoD
	R: CTGGGCTCCACTGTCACTCA	
MyHC	F: CTCCTCACGCTTTGGTAA	qRT-PCR for MyHC
	R: TGATAGTCGTATGGGTTGGT	
GAPDH	F: TCCTCCACCTTTGATGCG	qRT-PCR for GAPDH
	R: GTGCCTGGCTCACTCCTT	
β-actin	F: TTGTTGACAATGGCTCCGGT	qRT-PCR for β-actin
	R: AACCATCACACCCTGATGTCT	
U6	F: CAAGGACCCATCGTTCCACA	qRT-PCR for U6
	R: CCATTGGACACGCAGAATGC	

**Table 2:** Oligonucleotide sequences in this study.

Name	Nucleotide Sequences (5′-3′)
si-circMGA	AGAUAGAAAAGUUCGUGTT
si-FAP	GCTCTTACACTGGATGATT
miR-144-5p mimic	GGAUAUCAUCAUAUACUGUAAG
miR-144-5p inhibitor	GAAUGUCAUAUACUACUAUAGG

## Data Availability

The authors would like to express thanks to all the selfless assistance from all the lab members.

## Supplementary Materials

The following supporting information can be downloaded at: https://www.mdpi.com/article/10.3390/ani12070873/s1, File S1: The complete agarose gel electrophoresis images.
