# Supplementary figures and images for: CircMGA Depresses Myoblast Proliferation and Promotes Myotube Formation through miR-144-5p/FAP Signal
Source: Animals (Basel). 2022 Mar 30;12(7):873. doi: 10.3390/ani12070873 (PMC8996899; doi:10.3390/ani12070873)

**File S1.** The complete agarose gel electrophoresis images.

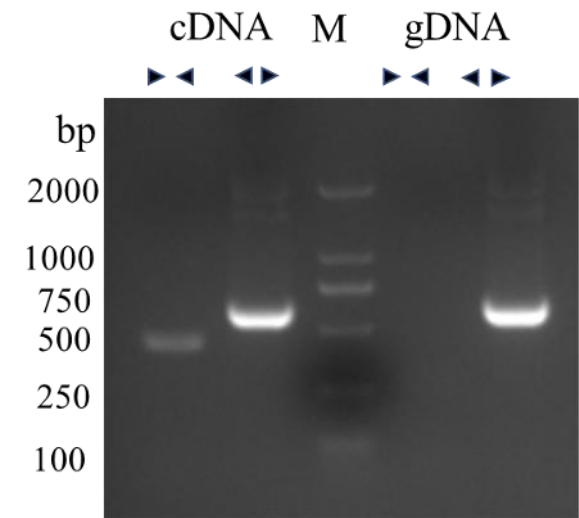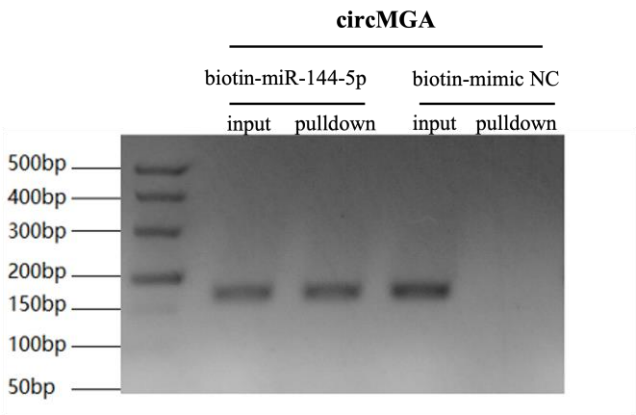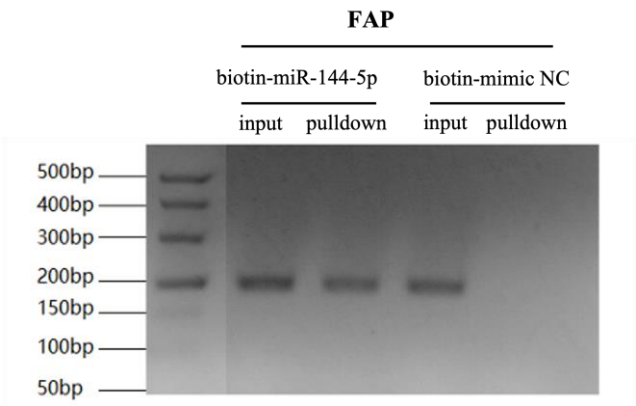

Supplement: Supplementary file 1 [file animals-12-00873-s001.zip › animals-1615328-supplementary.pdf]
